# Myeloid cell TNFR1 signaling dependent liver injury and inflammation upon BCG infection

**DOI:** 10.1038/s41598-019-41629-9

**Published:** 2019-03-28

**Authors:** Leslie Chavez-Galan, Dominique Vesin, Guillaume Blaser, Husnu Uysal, Sulayman Benmerzoug, Stéphanie Rose, Bernhard Ryffel, Valérie F. J. Quesniaux, Irene Garcia

**Affiliations:** 1 Department of Pathology and Immunology, Centre Medical Universitaire (CMU), Faculty of Medicine, University of Geneva, Geneva, 1211 Switzerland; 2 CNRS, UMR7355 Orleans, France; 3 Experimental and Molecular Immunology and Neurogenetics, University of Orléans, Orléans, France; 4 Laboratory of Integrative Immunology, National Institute of Respiratory Diseases “Ismael Cosio Villegas”, Mexico City, 14080 Mexico

## Abstract

TNF plays a critical role in mononuclear cell recruitment during acute *Bacillus Calmette-Guérin* (BCG) infection leading to an effective immune response with granuloma formation, but may also cause tissue injury mediated by TNFR1 or TNFR2. Here we investigated the role of myeloid and T cell specific TNFR1 and R2 expression, and show that absence of TNFR1 in myeloid cells attenuated liver granuloma formation and liver injury in response to acute BCG infection, while TNFR2 expressed in myeloid cells contributed only to liver injury. TNFR1 was the main receptor controlling cytokine production by liver mononuclear cells after antigenic specific response, modified CD4/CD8 ratio and NK, NKT and regulatory T cell recruitment. Further analysis of CD11b^+^CD3^+^ phagocytic cells revealed a TCRαβ expressing subpopulation of unknown function, which increased in response to BCG infection dependent of TNFR1 expression on myeloid cells. In conclusion, TNFR1 expressed by myeloid cells plays a critical role in mononuclear cell recruitment and injury of the liver after BCG infection.

## Introduction

*Bacillus Calmette-Guérin* (BCG) is a live attenuated *Mycobacterium bovis*, which is currently used as vaccine against *Mycobacterium tuberculosis* (*M*.*tuberculosis*) infection. BCG vaccination confers non-specific protection against non-related mycobacterial infections as reported by epidemiological studies showing better survival in BCG vaccinated children^1^. Recent studies have shown that vaccination with BCG protects from viral infections by mechanisms involving epigenetic reprograming of human monocytes and cytokine profile indicating trained immunity^2^. BCG vaccination can be detrimental in case of immunodeficiency such as in patients with chronic granuloma disease^3,4^. BCG promotes Th1 type immune responses and has been associated with a transient protective effect on asthma symptoms^5^. BCG, inducing regulatory T cells and eliminating autoreactive T cells through Tumor necrosis factor (TNF) activities, has been proposed as treatment for autoimmune diseases such as type 1 diabetes and multiple sclerosis^6^. BCG is used as immunotherapy of non-muscle-invasive bladder cancer by intravesical instillation^7^. Despite the low virulence of live BCG, under treatment few patients can develop adverse effects such as granulomatous hepatitis, probably due to the very high amount of bacilli used for this local therapy^8,9^.

BCG activates TNF production, which is one important proinflammatory cytokine required for protection against mycobacteria while TNF is also involved in mycobacterial-induced liver injury. TNF can function as bioactive membrane-bound form called transmembrane TNF (tmTNF), which is released as a soluble form (solTNF) by TNF-α converting enzyme (TACE). Both the solTNF as well as the tmTNF interact with TNFR1 and TNFR2. TNF protective immunity against mycobacteria is mainly mediated through TNF receptor 1 (TNFR1) while TNF receptor 2 (TNFR2) is involved in tolerogenicity^10–14^. Our previous studies have shown that TNFR1 expressed by myeloid cells is the first line of defense against *M*. *tuberculosis* infection as myeloid cells deficient in TNFR1 recapitulates the phenotype of total TNFR1 KO mice^14^. We have also shown that tmTNF, expressed by myeloid-derived suppressor cells (MDSC) interacting with CD4 T cells expressing TNFR2, mediates tolerogenic activity and controls the exacerbated inflammation during acute mycobacterial-induced pleurisy^15^. However, during chronic *M*. *tuberculosis* infection, TNF interaction with TNFR2 can be detrimental illustrating the complexity of the TNF system^13^.

BCG induces granuloma formation in infected organs and cell activation. Previous data have shown that neutralization of TNF and gene deletion prevents cell recruitment and impairs BCG granuloma formation^16–18^. While TNF is required for granuloma formation and protection, its high expression during acute infection may cause tissue damage. In particular, in hepatic cell damage with increased serum transaminase levels is a common finding. We have reported that only solTNF but not tmTNF mediates BCG-induced liver injury using both genetic and pharmacologic approaches^18^. However, the importance of TNF receptors as well as their cell specific expression is unknown.

To investigate how the absence of TNFR1 or TNFR2 expression on myeloid and lymphoid cells influences liver cell recruitment during acute BCG infection and their potential hepatotoxicity, we have used a genetic approach with mice bearing a specific deletion of TNFR1 on myeloid (TNFR1-M KO) or on T cells (TNFR1-T KO). In addition, to explore the role of myeloid or lymphoid cells expressing TNFR2, we have also used mice with deletion of TNFR2 on myeloid (TNFR2-M KO) or on T cells (TNFR2-T KO). Here, we show that liver cell recruitment in response to BCG-infection is mainly controlled by TNFR1. TNFR1 deficiency affects the recruitment of both myeloid and lymphoid cells, including the presence and activity of CD3^+^ myeloid cells already described in BCG granulomas^19^. In contrast, myeloid or lymphoid TNFR2 depletion affects marginally hepatic cell recruitment but causes changes in cell function during BCG infection. Interestingly, myeloid cells expressing either TNFR1 or TNFR2 contribute to liver injury.

## Results

### Inflammatory status and hepatotoxicity after BCG infection are mediated mainly by myeloid cell TNFR1

**Figure 1:**
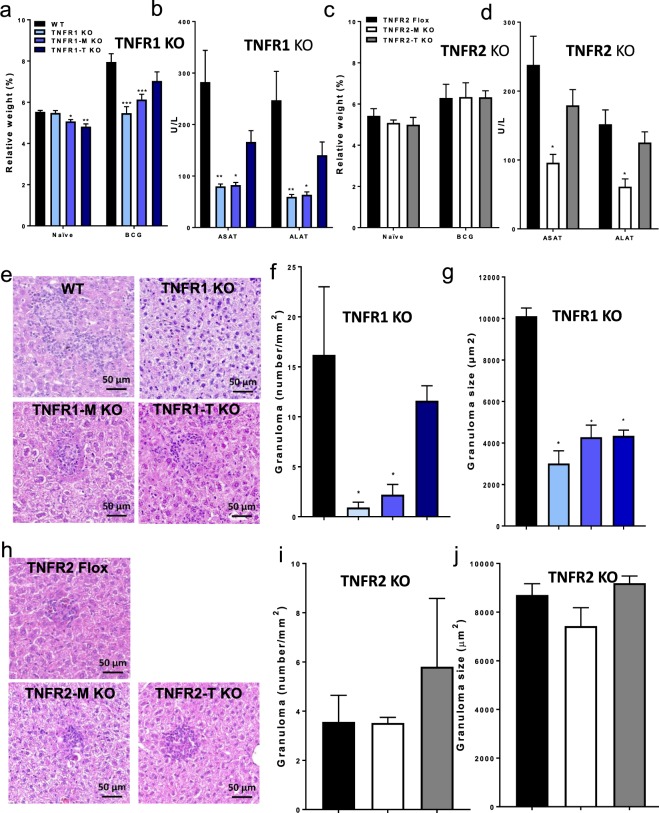
Myeloid cell TNFR1 controls the inflammatory status and hepatotoxicity after BCG infection. (**a**) Relative liver weight in naïve littermates and at 2-weeks post-infection of: WT, TNFR1 KO, TNFR1-M KO, TNFR1-T KO and (**c**) TNFR2-Flox, TNFR2-M KO and TNFR2-T KO. Serum AST/ALT measured at 2-weeks post-infection in WT, TNFR1 KO, TNFR1-M KO, TNFR1-T KO or in TNFR2-Flox, TNFR2-M KO and TNFR2-T KO (**b**,**d** respectively). (**e**,**h**, respectively) Microscopic examination of liver from mice at 2-weeks post-infection, in total or partial absence of TNFR1 and TNFR2 mice. (**f**,**g**, respectively) The number and size of granulomas in WT, TNFR1 KO, TNFR1-M KO, TNFR1-T KO and (**i**,**j**, respectively) TNFR2-Flox, TNFR2-M KO and TNFR2-T KO mice were assessed using ZEISS ZEN 2 imaging-software. Results are representative of three independent experiments. Bar graphs shown mean +/− SEM (**a**–**d**,**f**,**g**,**i**,**j**). n = 3 to 9 mice per group. *p < 0.05, **p < 0.01, ***p < 0.001 versus WT, multiple t test and Bonferroni-Dunn were used to multiple comparison. Scale bar = 50 µm.

To assess the relative contribution of the cell specific TNFRs expression on cell recruitment to the liver during the early responses to intravenous BCG infection, WT, TNFR1 KO, TNFR1-M KO, TNFR1-T KO, TNFR2 Flox, TNFR2-M KO and TNFR2-T KO mice were infected with living BCG and liver analyzed at 2-weeks post-infection. Relative liver weight is a first indicator of liver inflammation in BCG-infected mice. At 2-weeks post-infection, TNFR1 KO and TNFR1-M KO but not TNFR1-T KO showed lower liver relative weight than WT mice, suggesting less inflammation, (Fig. 1a). Liver relative weight of TNFR1-M KO mice correlated with the reduced serum levels of aspartate and alanine transaminases (AST and ALT, respectively) (Fig. 1b). However, the total number of CFU in the liver was not statistically different between phenotypes at this time point of the infection (data not shown). In contrast, TNFR2 Flox, TNFR2-M KO and TNFR2-T KO mice showed similar increase in relative liver weight after BCG infection (Fig. 1c) and surprisingly AST and ALT levels were lower in TNFR2-M KO (Fig. 1d). Liver histopathologic examination revealed that the number and size of granulomas were lower in TNFR1 KO and TNFR1-M KO compared to WT mice (Fig. 1e–g). Cell specific deficiency of TNFR2 did not influence significantly granuloma number and size as compared to TNFR2 Flox mice (Fig. 1h–j). These data show that after BCG-infection, TNFR1 on myeloid or lymphoid cells plays a predominant role to control both liver inflammation and granuloma formation, but TNFR2 expressed on myeloid cells only contributes to hepatotoxicity. These data suggest that the role of TNFRs on myeloid cells is fundamental to induce hepatotoxicity but TNFR1 also controls granuloma formation.

### Cell specific deletion of TNFR1 or TNFR2 impairs ex-vivo cytokine release after BCG stimulation of liver mononuclear cells

**Figure 2:**
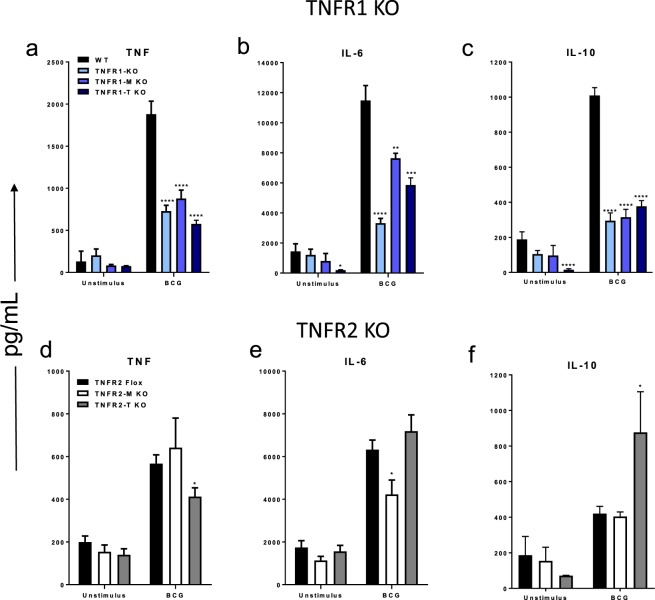
Absence of TNFR1 and TNFR2 affects the ability of liver mononuclear cells (LMC) to produce cytokines in response to antigen stimulation. LMCs from 2-weeks infected, *in vitro* infected with BCG MOI 1 for 24 h. (**a**–**c**, respectively), TNF, IL-6 and IL-10 were assessed in WT, TNFR1 KO, TNFR1-M KO, TNFR1-T KO and (**d**,**e** and **f**, respectively) TNF, IL-6 and IL-10 were assessed in TNFR2-Flox, TNFR2-M KO and TNFR2-T KO. Results are representative of three independent experiments. Bar graphs shown mean +/− SEM (**a**,**c**). n = 3 to 7 mice per group. *p < 0.05, **p < 0.01, ***p < 0.001, ****p < 0.0001 versus WT, multiple t test and Bonferroni-Dunn were used to multiple comparison.

To evaluate if liver mononuclear cells (LMCs) from mice with cell specific deletion of TNFR1 or TNFR2 can respond to an antigenic stimulation, LMCs from 2-weeks BCG-infected mice were isolated and their cytokine response assessed. BCG stimulation of LMCs from infected WT mice activated high levels of TNF, interleukin (IL)-6 and IL-10 (Fig. 2a–c). However, total or cell specific deletion of TNFR1 on myeloid or on lymphoid cells altered the responses of TNF, IL-6 and IL-10 which were lower than WT cells (Fig. 2a–c). In contrast, myeloid cell specific depletion of TNFR2 affected the expression of IL-6 whereas lymphoid TNFR2 depletion resulted in lower levels of TNF but higher IL-10 indicating the cell specific expression of TNFR2 is necessary for control of these two cytokines. (Fig. 2d–f). These data suggest that activation of TNF, IL-6 and IL-10 depends on both myeloid and lymphoid cell TNFR1. However, IL-6 activation only depends on myeloid cell TNFR2 while the control of the balance between TNF and IL-10 depends on lymphoid cell TNFR2.

### Myeloid cell TNFR1 affects T cell recruitment and TNFR2 regulates Treg function

We further study the phenotype of lymphoid cell populations included in LMCs in uninfected (naïve) mice and at two weeks post-BCG infection. Previous reports have shown that during a BCG infection, CD3 expression is not restricted to T cells in the liver of infected mice. Indeed, CD3 is expressed by both myeloid and lymphoid subpopulations that can also bear a specific TCR chain^19^. Thus, in order to be stricter in the identification of CD4 and CD8 T cells, we defined first the percentage of CD3^+^TCRαβ^+^ cells (T cells) and then, the expression of CD4 (CD4 T cells) and CD8 (CD8 T cells) was evaluated in this gate (see Supplementary Fig. S1a). The frequency of CD4 T cells did not change at 2 weeks post-infection independently of mouse genetic background, while the frequency of CD8 T cells was already lower in deficient genotypes compared to WT both before and after infection (see Supplementary Fig. S1b,c). In contrast, TNFR2-M and TNFR2-T KO mice did not show any changes in the frequency of CD4 or CD8 T cells compared to controls TNFR2 flox mice (see Supplementary Fig. S1d,e). Our data suggest that myeloid and lymphoid cell TNFR1 play an important role to maintain the CD4/CD8 balance in the liver before and after BCG infection, while TNFR2 does not alter CD4/CD8 ratio.

**Figure 3:**
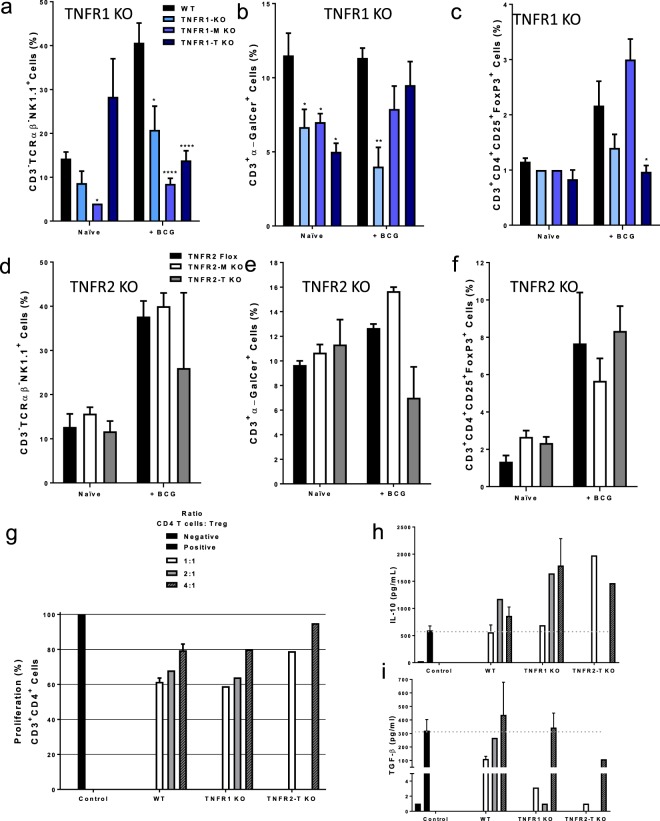
Depletion of TNFR1 affects the recruitment of lymphoid cells in the liver during BCG infection, but the Tregs function is mediated by TNFR2. LMC from WT, TNFR1 KO, TNFR1-M KO, TNFR1-T KO, TNFR2-Flox, TNFR2-M KO and TNFR2-T KO were obtained and prepared for flow cytometry. (**a**) The frequency of NK cells (CD3-TCRαβ-NK1.1+), (**b**) of NKT cells (CD3+ α-GalCer+), and (**c**) of Treg cells (CD3+ CD4+ CD25+ FoxP3+) on deficient of TNFR1 is reported. (**d**) The frequency of of NK cells (CD3-TCRαβ-NK1.1+), (**e**) of NKT cells (CD3+ α-GalCer+), and (**f**) of Treg cells in TNFR2 deficient mice is reported. Results are representative of three independent experiments. Bar graphs shown mean +/− SEM, n = 3 to 9 mice per group. *p < 0.05, **p < 0.01 ***p < 0.001 versus WT, Multiple t test and Bonferroni-Dunn were used to multiple comparison. (**g**) Treg cells from WT, TNFR1 KO and TNFR2-T KO mice at 2-weeks infection were purified, and put in co-culture with CD4 T cells enrichment (from WT) in a ratio 1:1, 2:1, 4:1 (CD4 T cell:Treg). Cells were stimulated with CD3/CD28/beads in a ratio 1:2 (CD4+ T cell:bead) by 72 h, proliferation was measured by flow cytometry by evaluation of the Marker KI-67. Positive control is CD4+ T cells with CD3/CD28/beads (without Treg), this value was considered 100% proliferation. (**h**,**i**, respectively) Supernatant was recovered, IL-10 and TGF-β were measured by ELISA Data are representative of 1 experiments by duplicate. Bar graphs shown mean +/− SEM, to purify Treg a pool of 2 WT, 4 TNFR1 KO and 3 TNFR2-T KO mice were used.

We also analyzed liver recruitment of Natural killer (NK) and natural killer T (NKT) cells which are subsets of lymphoid cells that express specific markers. NK cells are identified by the phenotype CD3^−^NK1.1^+^ while NKT cells are CD3^+^ but they express a specific TCR highly reactive to the glycolipid alpha galactosylceramide (αGalCer)^20,21^. We assessed the frequency of NK cells by gating CD3^−^TCRαβ^−^ cells and then NK1.1^+^ expression. Liver from naïve TNFR1-M KO mice displayed lower frequency of NK cells compared to WT mice. BCG infection showed that both total and cell specific deficiency of TNFR1 affected NK recruitment to the liver (Fig. 3a). In contrast, cell specific TNFR2 deficiency did not change the frequency of NK cells (Fig. 3d). NKT cells were identified as CD3^+^ cells also reacting with the αGalCer tetramer in the CD1d complex (αGalCer/CD1d) as previously described^22^. Thus, CD3^+^αGalCer/CD1d^+^ cells are NKT cells (see Supplementary Fig. S2b). Naïve TNFR1-KO, TNFR1-M and TNFR1-T KO mice showed a lower frequency of NKT cells, however at 2-weeks after infection only TNFR1 KO mice maintained the deficiency of NKT because mice with partial deletion of TNFR1 recovered the ability to recruit this cell subpopulation (Fig. 3b). In contrast, TNFR2 deletion did not affect liver NKT recruitment (Fig. 3e). Previous reports have shown that TNFR2 promotes the expansion and function of Tregs, which exhibit a CD3^+^CD4^+^CD25^+^FoxP3^+^ phenotyp^23^. The frequency of Tregs was evaluated by the co-expression of CD25 and FoxP3 on gated CD3^+^CD4^+^ cells (see Supplementary Fig. S2c). TNFR1-T KO but not TNFR1-M KO mice exhibited a lower frequency of Tregs compared to WT in the liver after infection (Fig. 3c). Surprisingly, absence of TNFR2 on myeloid or in lymphoid cells did not influence the recruitment of Tregs (Fig. 3f). To further characterize whether TNFR1 or TNFR2 influence Treg function, Tregs from WT, TNFR1 and TNFR2-T KO were isolated and co-cultured with CD4 T cells, and cellular proliferation and cytokine production evaluated. Our data showed that co-cultures of Tregs from WT and TNFR1 KO reduced the proliferation of CD4 T cells by 40% (Fig. 3g). However, Tregs from TNFR2-T KO were less efficient in inhibiting CD4 T cell proliferation (Fig. 3g). IL-10 and TGF-β has been involved as one of the mechanism of Treg-mediated suppression^24^. TNFR2-T KO Tregs induced an imbalance in IL-10 and TGF-β production as compared to WT, in the ratio 1:1 was characterized by 3 times higher IL-10 while TGF-β was not produced (Fig. 3h,i). Together these data indicated that TNFR1 is the main receptor to control the lymphoid cell recruitment to the liver during BCG infection; however, TNFR2 is necessary for Treg suppressive function, probably by a TGF-β dependent-mechanism.

### Myeloid and lymphoid cell TNFR1 as well as lymphoid cell TNFR2 control macrophage recruitment to the liver after BCG infection

**Figure 4:**
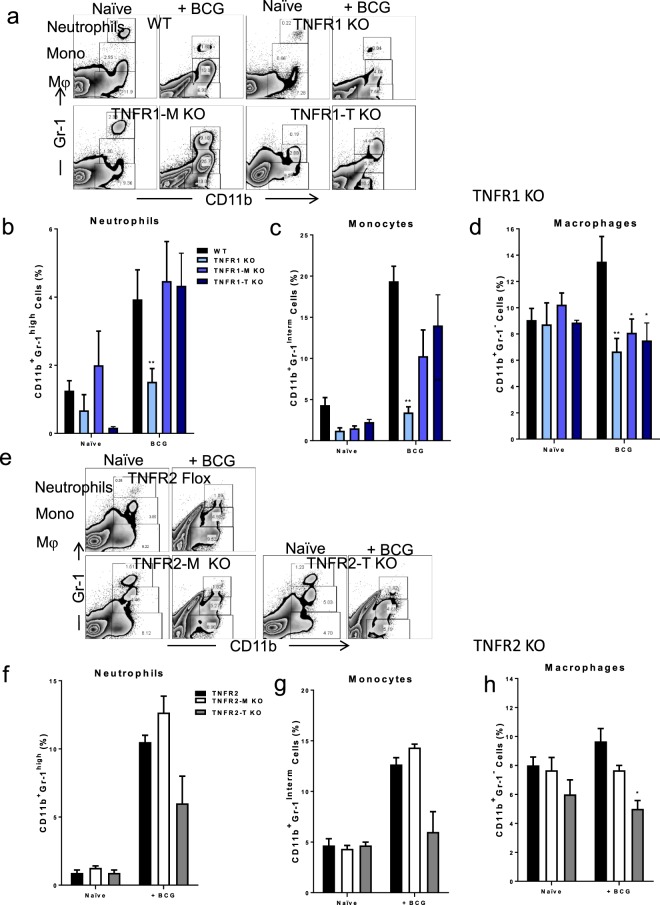
Macrophage recruitment to the liver is modified by cell specific deficiency of TNFR1 after BCG infection. (**a**) Representative zebra plot showing liver cells in naïve littermates and at 2-weeks post-infection, liver myeloid subsets were identified by CD11b and Gr-1 expression from LMC obtained from WT, TNFR1 KO, TNFR1-M KO, and TNFR1-T KO mice. (**b**) Frequency of Neutrophils (CD11b^+^Gr-1^high^), (**c**) Monocytes (CD11b^+^Gr-1^Interm^) and (**d**) Macrophages (CD11b^+^Gr-1^−^) is shown. (**e**) Representative zebra plot showing liver cells in naïve littermates and at 2-weeks post-infection, liver myeloid subsets were identified as in (**a**) with LMC obtained from TNFR2-Flox, TNFR2-M KO and TNFR2-T KO. (**f**) Frequency of Neutrophils (CD11b^+^Gr-1^high^), (**g**) Monocytes (CD11b^+^Gr-1^Interm^) and (**h**) Macrophages (CD11b^+^Gr-1^−^) is shown. Results are representative of three independent experiments. Bar graphs shown mean +/− SEM, n = 3 to 9 mice per group (B and C). *p < 0.05, **p < 0.01, ***p < 0.001 versus WT, Multiple t test and Bonferroni-Dunn were used to multiple comparison.

We examined liver myeloid cell subsets based on CD11b and GR-1 markers as previously described^25^. Using myeloid CD11b and GR-1 markers, CD11b^+^Gr-1^high^ cells were considered as neutrophil, CD11b^+^Gr-1^Interm^ as monocytes and CD11b^+^Gr-1^−^ as macrophages (Fig. 4a–d). Our data showed that TNFR1 KO mice showed impaired recruitment of neutrophils, monocytes and macrophages (Fig. 4b–d). However, TNFR1-M and TNFR1-T only exhibited a significant decrease in macrophage recruitment (Fig. 4b–d). In contrast, only TNFR2-T KO mice showed lower recruitment of macrophages (Fig. 4e–h). We further evaluated in each subpopulation the co-expression of Ly6C, F4/80 and CD11c to confirm the phenotype of myeloid cells and observed no important difference between WT and TNFR1 KO cells, with the exception of lower expression of CD11c in the monocyte subpopulation of TNFR1 KO compared to WT cells (see Supplementary Fig. S3a–c). These data suggest that partial expression of TNFR1 is enough to rescue neutrophils and monocytes, however, macrophage recruitment is highly dependent on TNFR1 expression.

### Myeloid and lymphoid cell TNFR1, but lymphoid TNFR2 influence MHC-II^+^ expression on myeloid cells after BCG infection

**Figure 5:**
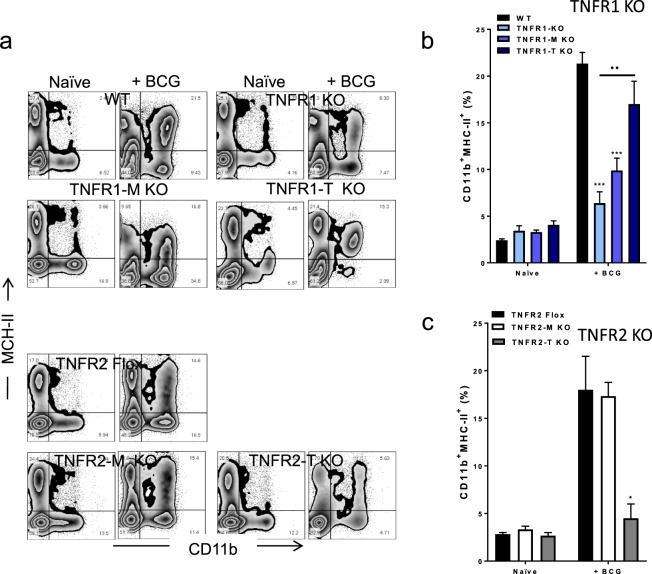
Deficiency of TNFR1 and TNFR2 may affect expression of MHC-II+ on myeloid cells after BCG infection. (**a**) Representative zebra plot showing LMC from WT, TNFR1 KO, TNFR1-M KO, TNFR1-T KO (up) and TNFR2-Flox, TNFR2-M KO and TNFR2-T KO (down) of LMC before and after infection. Liver cells were evaluated by flow cytometry using CD11b and MHC-II expression markers. (**b**) The frequency of CD11b^+^MHC-Ii^high^ in TNFR1 deficient and (**c**) in TNFR2 deficient mice is reported. Results are representative of three independent experiments. Bar graphs shown mean +/− SEM, n = 3 to 9 mice per group (**b**,**c**). *p < 0.05, **p < 0.01, ***p < 0.001 versus WT. ••p < 0.05 TNFR1 KO versus TNFR1-M KO and TNFR1-T KO, Multiple t test and Bonferroni-Dunn were used to multiple comparison.

Activation of the adaptive immune system requires the antigen presentation of microbial antigenic peptides by Major Histocompatibility Complex Class II (MHC-II), molecule expressed by antigen-presenting cells^26^. We have evaluated whether MHC-II expression on myeloid cells (based on CD11b expression) can be affected by cell specific deficiency on TNFR1 or on TNFR2 expression. Cells expressing CD11b^+^MHC-II^+^ were identified by flow cytometry (Fig. 5a). We found that total deletion of TNFR1 dramatically affected the frequency of CD11b^+^MHC-II^+^ cells after BCG infection (Fig. 5b). However, TNFR1-M and TNFR1-T KO mice partially rescued the frequency of liver CD11b^+^MHC-II^+^ cells compared to WT and TNFR1 KO mice (Fig. 5b). TNFR2-T KO but not TNFR2-M KO mice showed a reduced frequency of liver CD11b^+^MHC-II^+^ cells (Fig. 5c). In summary, total and cell specific TNFR1 deletion affects the recruitment of CD11b^+^MHC-II^+^ cells, which was also found on T cell TNFR2 deficient mice.

### BCG infection induces liver recruitment of CD3^+^ TCRαβ^+^/TCRαβ^−^ myeloid cells

**Figure 6:**
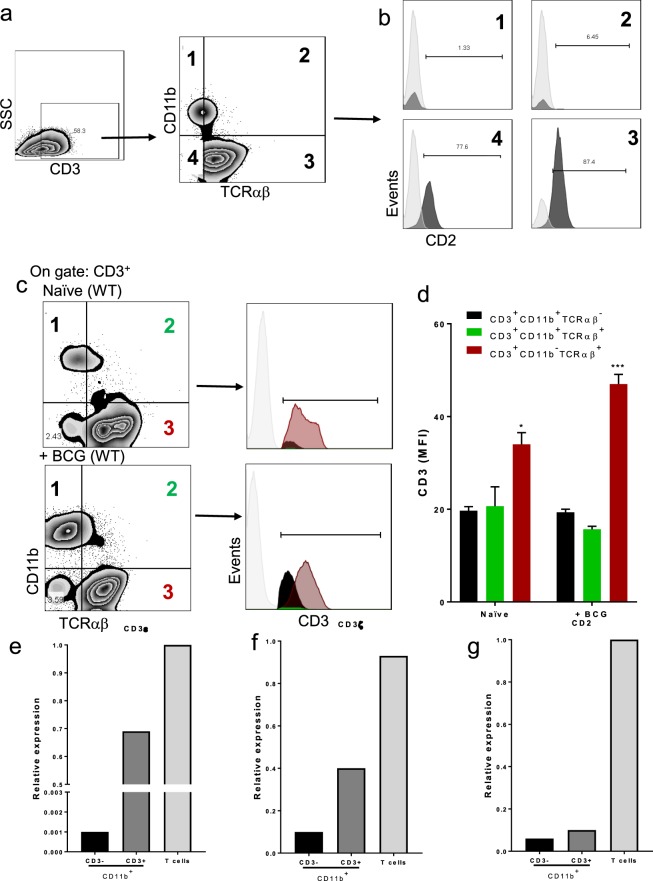
CD3^+^CD11b^+^TCRαβ^−^ and CD3^+^CD11b^+^TCRαβ^+^ myeloid cells identified at the protein and transcriptional levels. LMC from WT were obtained and prepared for flow cytometry or sorted to perform qPCR. (**a**) Representative zebra plot showing LMC from infected mice, CD3^+^ gate was delimited, and co-expression of CD11b and TCRαβ was evaluated, and 4 subpopulations were identified. (**b**) Inside each subpopulation CD2 expression was evaluated. Data are from 3 independent animals. (**c**) Representative zebra plot of LMC from naïve littermates (up) and after 2-weeks infection (down), the subpopulations CD3^+^CD11b^+^TCRαβ^−^, CD3^+^CD11b^+^TCRαβ^+^ and CD3^+^CD11b^−^TCRαβ^+^ and CD3^+^CD11b^+^TCRαβ^+^ were identified to evaluate the mean fluorescence intensity (MFI) of CD3, histograms display CD3 MFI (**d**), bar graphs shown mean +/− SEM, n = 3 mice per condition. **p < 0.01, ***p < 0.001 versus CD3^+^ myeloid cells. Multiple t test and Bonferroni-Dunn were used to multiple comparison. -Pooled LMC (3 mice) from WT, CD3^−^CD11b^+^ and CD3^+^CD11b^+^ were sorted by flow cytometry. In purified subpopulations (**e**) CD3ε, (**f**) CD3ξ and (**g**) CD2 relative gene expression was evaluated by real-time PCR CD4+ T cells isolated were used as a positive control.

Recent experimental evidence revealed a myeloid cell subpopulation involved in BCG granuloma formation expressing the complex CD3/TCR, and TCR β-chain expression on macrophages which is TNF-dependent^19^. To evaluate the presence of these cells, we isolated LMCs from the liver of WT naïve mice at 2-weeks post- BCG and followed the analysis strategy represented in Fig. 6a. First, CD3^+^ cells were gated and then analyzed for the co-expression of CD11b and TCRαβ. Unexpectedly, we identified two CD3^+^ myeloid subpopulations, one more abundant and phenotypically TCRαβ^−^ and a second TCRαβ^+^ (Fig. 6a). Then, four subpopulations were identified: Two of CD3^+^ myeloid cells (CD3^+^CD11b^+^TCRαβ^−^ and CD3^+^CD11b^+^TCRαβ^+^), one of classical T (CD3^+^CD11b^−^TCRαβ^+^) and one of no-classical T cells (NKT or T cells TCRγδ) (CD3^+^CD11b^−^TCRαβ) (Fig. 6a). To discard the possibility that CD3^+^CD11b^+^TCRαβ^−^ and CD3^+^CD11b^+^TCRαβ^+^ subpopulations were lymphoid cells, we assessed the expression of CD2 as a lymphoid marker in each one of the four subpopulation. As we expected, CD3^+^ myeloid cells (CD11b^+^) were negative for CD2, while the CD3^+^CD11b^−^ were clearly CD2^+^ (Fig. 6b). Then, we examined if CD3^+^ myeloid cells have the same intensity of CD3 expression compared to lymphoid cells, by assessing the mean fluorescence intensity (MFI) of CD3 cells (Fig. 6c). Our results showed that myeloid cells expressed lower CD3 MFI than lymphoid cells. Interestingly, after BCG infection lymphoid cells increased CD3 MFI expression but no myeloid cells, suggesting that activated myeloid cells are low CD3 expressing cells (Fig. 6d). Another possibility is that there are CD3^+^ myeloid cells due to endocytosis or phagocytosis of endosomes delivered by T cells and consequently myeloid cells are CD3^+^. To exclude this possibility, myeloid cells (CD11b^+^) CD3^+^ and CD3^−^ were sorted by flow cytometry, and CD3ε (epsilon chain) and CD3ζ (zeta chain) expression were evaluated at the transcription level by real-time PCR, and isolated CD4 T cells used as positive control for the expression of CD3 and CD2 (Fig. 6e–g). Our result showed that both CD3ε and CD3ζ genes were expressed in CD3^+^CD11b^+^ myeloid cells while CD3^−^CD11b^+^ were negative (Fig. 6e,f). Moreover, we assessed the expression of the CD2 gene in isolated T cells compared to CD3^+^CD11b^+^ cells to exclude that these cells are lymphoid cells. Our qPCR data confirmed that CD2 is not expressed at the transcription level in CD3^+^CD11b^+^ and CD3^−^CD11b^+^ cells which is agreement with its absence at the protein level observed by flow cytometry (Fig. 6g). These results together confirm that, considering CD3 expression, there are two myeloid subpopulation CD3^+^CD11b^+^TCRαβ^−^ and CD3^+^CD11b^+^TCRαβ^+^ which do not express CD2, suggesting that these cells are not lymphoid cells.

We further evaluated whether BCG infection can regulate the expression of lymphocyte proteins and genes in a macrophage murine cell line (RAW cells). We assessed the expression of CD3 by FACS, western blot and qPCR analyses in RAW cells infected with BCG at different time points. We found that BCG infection upregulated the expression of CD3 in a time dependent manner (see Supplementary Fig. S4a). Western analyses confirmed the expression of CD3ε which increased with time of infection (see Supplementary Fig. S4b,c). Our qPCR data showed upregulation of both CD3ε and CD3ζ genes at 2 hours which declined at 5 and 24 hours post-infection (see Supplementary Fig. S4d).

### Myeloid cell TNFR1 controls the liver recruitment of CD3^+^ TCRαβ^+^/TCRαβ^−^ myeloid cells after BCG infection

**Figure 7:**
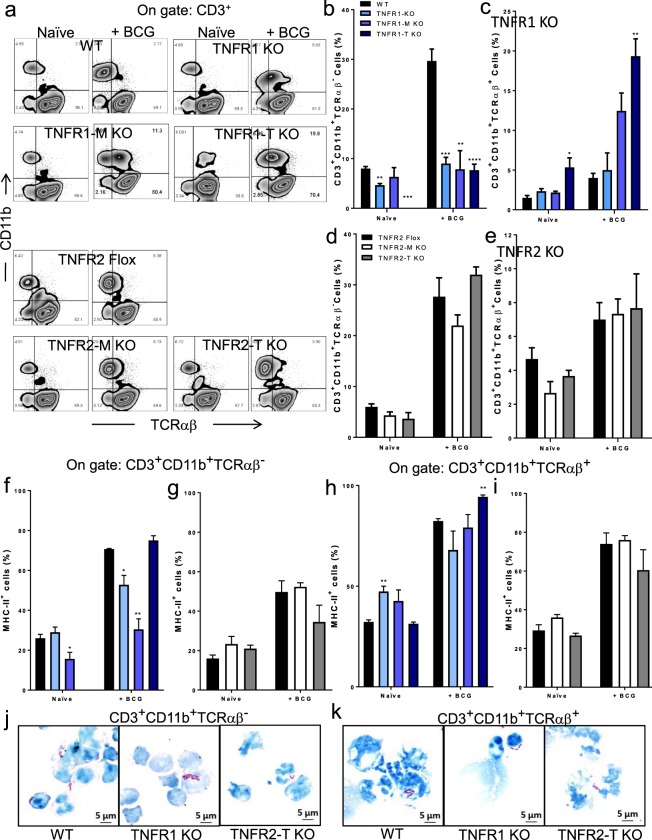
TNFR1 plays a main role to recruit CD3+ CD11b+ TCRαβ- and CD3+ CD11b+ TCRαβ+ cells in BCG-infected mice. (**a**) Representative zebra plot showing LMC from naïve littermates and after infection from (up) WT, TNFR1 KO, TNFR1-M KO, TNFR1-T KO, and (down) TNFR2-Flox, TNFR2-M KO and TNFR2-T KO mice. A first gate of CD3^+^ cells was done and inside this gate, CD11b and TCRαβ expression was evaluated. (**b**) The frequency of CD3^+^CD11b^+^TCRαβ^−^ and (**c**) CD3^+^CD11b^+^TCRαβ^+^ in WT and TNFR1 deficient mice or in (d and e) TNFR2-Flox, TNFR2-M KO and TNFR2-T KO mice is shown. (**f**,**g**) Inside the gate of CD3^+^CD11b^+^TCRαβ^−^ and (**h**,**i**) CD3+ CD11b+ TCRαβ+ the co-expression of MHC-II^+^ was evaluated both TNFR1 and TNFR2 deletion. Results are representative of three independent experiments. Bar graphs shown mean +/− SEM, n = 3 to 5 mice per group. *p < 0.05, **p < 0.01, ***p < 0.001, ****p < 0.0001 versus WT. •••p < 0.001 between groups indicated, Multiple t test and Bonferroni-Dunn were used to multiple comparison. (**j**) Pool of LMC from WT (2 mice), TNFR1 KO (4 mice) and TNFR2-T KO (3 mice), after 2-weeks infection and CD3^+^CD11b^+^TCRαβ^−^ and (**k**) CD3^+^CD11b^+^TCRαβ^+^ after sorting by flow cytometry, cytospin were prepared and cells were stained with Ziehl-Neelsen, Photomicrographs from cytospin preparation show intracellular bacilli (red).

We then asked if both CD3^+^CD11b^+^TCRαβ^−^ and CD3^+^CD11b^+^TCRαβ^+^ myeloid subpopulations increase as response to BCG infection and if TNFR1 or TNFR2 may influence the presence of these subpopulations. Following the analysis strategy represented in Fig. 7a, we identified again both subpopulations of CD3+ myeloid cells in the liver of mice with different genotypes and their frequency increased in response to BCG infection. TNFR1 deficient mice showed lower frequency of CD3^+^CD11b^+^TCRαβ^−^ cells compared with WT mice. However, BCG infection induced a 3-folds increase in the frequency of CD3^+^CD11b^+^TCRαβ^−^ cells in WT which was not found in TNFR1 KO as well as in TNFR1-M and TNFR1-T KO mice (Fig. 7b). Regarding the frequency of CD3^+^CD11b^+^TCRαβ^+^ cells, naïve TNFR1-T KO mice showed an increase compared to WT mice (Fig. 7c). After infection, all mouse genotype increased the frequency of CD3^+^CD11b^+^TCRαβ^+^ but TNFR1-T KO mice showed stronger increase (Fig. 7c). Cell specific deletion of TNFR2 did not change the frequency of both CD3^+^CD11b^+^TCRαβ^−^ and CD3^+^CD11b^+^TCRαβ^+^ subpopulations (Fig. 7a down, d-e).

To explore if CD3^+^CD11b^+^TCRαβ^−^ and CD3^+^CD11b^+^TCRαβ^+^ myeloid cells can have a functional activity such as antigen presentation, we evaluated MHC-II co-expression in both subpopulation. MHC-II increased in these two cell subpopulations with BCG infection (Fig. 7f-i). However, TNFR1 KO and TNFR1-M KO infected mice showed lower frequency of CD3^+^CD11b^+^TCRαβ^−^ co-expressing MHC-II (Fig. 7f). While the frequency of CD3^+^CD11b^+^TCRαβ^+^MHC-II^+^ was higher in TNFR1-T KO infected mice (Fig. 7h). Our data also showed that TNFR2 deletion had no effect on the frequency of liver CD3^+^CD11b^+^TCRαβ^-^MHC-II^+^ and CD3^+^CD11b^+^TCRαβ^+^MHC-II^+^ subpopulations (Fig. 7h,i).

As TNFR1 deletion affects the recruitment and the expression MHC-II of CD3^+^CD11b^+^TCRαβ^−^ and CD3^+^CD11b^+^TCRαβ^+^ cells, we hypothesized that the function of these subpopulations could also be affected by TNFR1 deficiency. In order to clarify this, both CD3^+^CD11b^+^TCRαβ^−^ and CD3^+^CD11b^+^TCRαβ^+^ subpopulations were sorted by flow cytometry, microscopic and functional analysis were performed. Purified CD3^+^CD11b^+^TCRαβ^−^ and CD3^+^CD11b^+^TCRαβ^+^ cells showed a morphology similar to monocyte/macrophages (see Supplementary Fig. S6a,b). Additionally, we observed that both cell subpopulations contain acid fast bacilli, as visualized by Ziehl-Neelsen staining and this phagocytic function is maintained independently of TNFR1 or TNFR2 deletion (Fig. 7j,k). CD3^+^CD11b^+^TCRαβ^−^ and CD3^+^CD11b^+^TCRαβ^+^ subpopulations also were able to respond to a BCG re-stimulation *in vitro* by delivering of cytokines such as TNF, IL-6 and IL-10 (see Supplementary Fig. S6c–e).

## Discussion

TNF is a master cytokine controlling host immunity against mycobacterial infections. While TNF is required for host defense against mycobacteria, TNF also has detrimental effects and generates inflammation and tissue damage during infections, particularly in the liver. Severe inflammatory disease can be treated with anti-TNF and compromise host immunity, as previously shown in animal models of tuberculosis^27–29^. However, based on the role of TNF in granuloma formation and maintenance, adjunctive therapy with anti-TNF during chemotherapy was shown to improve bacterial clearance and lung pathology^30,31^. TNF activities are complex, however a deeper understanding of TNF signaling may provide strategies to develop new therapeutic approaches.

As TNF can be expressed as two molecular forms, several approaches to decipher specific activities of tmTNF versus solTNF have been developed. We have previously shown that during mycobacterial infections solTNF but not tmTNF mediates liver toxicity but tmTNF protects host against mycobacterial infections^32–34^. In several models of hepatitis mediated by TNF, it has been shown that only solTNF promotes liver necrosis but not tmTNF^18^. This insight leaded to novel approach of host-directed therapy proposing selective neutralization of solTNF sparing tmTNF by dominant-negative TNF molecules, which was shown to preserve host immunity against acute tuberculosis infection^18^.

To go further in the understanding of the TNF pathways, the analysis of cells interacting with tmTNF expressing cells has been carried out using a mouse model of BCG-induced pleurisy^35^. MDSC expressing tmTNF were shown to interact with T cells expressing TNFR2 and to attenuate the inflammatory process associated with BCG-induced pleurisy in mice, suggesting that tmTNF/TNFR2 is an important pathway to regulate the T cell proliferation and subsequently decrease the inflammation^15^. However, as TNFR1 plays an essential role for host defense against mycobacteria, we have studied TNFR1 specific expression on myeloid versus lymphoid cells during tuberculosis infection^10,14^. We have reported that specific expression of TNFR1 on macrophages and neutrophils is required for resistance against tuberculosis, but expression on T cells is dispensable^14^.

We have now studied the role of cell specific expression of TNFR1 and TNFR2 in the cell recruitment to the liver and hepatotoxicity following BCG infection in mice. Our results show that: (1) TNFR1 expressed by myeloid cells is the predominant receptor influencing cell recruitment, MHC-II expression, and liver necrosis during an acute infection by BCG. In contrast, myeloid cell TNFR2 only affects lightly liver cell recruitment but surprisingly influences liver toxicity. (2) TNFR1 controls the levels of cytokines reactivated by BCG on MLCs. (3) TNFR1 but not TNFR2 controls the balance of liver CD4/CD8 T cells. (4) TNFR1 expression regulates liver recruitment of NK1.1 cells. (5) TNFR1 on T cells controls Treg recruitment but TNFR2 regulates suppressive activity. (5) Myeloid and lymphoid cell TNFR1 but only lymphoid cell TNFR2 control liver recruitment of macrophages and MHC-II activation. (6) TNFR1 regulates the presence of a specific myeloid subpopulation, characterized as CD11b+ CD3+ TCR− cells, interestingly the MHC-II expression on CD3+ myeloid cells also was modified by TNFR1.

Together these data confirm that TNFR1 is the main receptor for transmembrane and soluble TNF regulating liver cell recruitment and liver injury after BCG infection (see Supplementary Fig. S7). Absence of TNFR1 on myeloid cells affected granuloma formation leading to decreased hepatotoxicity. This is interesting to note that the size of hepatic granuloma, but not their number, was found smaller in TNFR1-T KO than in WT. It can be due to an impaired cell recruitment at this time point of the infection. This result suggests that TNFR1 deficiency in T cells affects cell recruitment or probably cell kinetics. However, granuloma are functional and mycobacteria can be eliminated in TNFR1-T but not in TNFR1-M KO mice as we have previously reported^14^. In contrast, absence of TNFR2 on myeloid cells did not show differences on granuloma formation but also resulted in reduced liver toxicity. These suggest that myeloid TNFR1 influence the number and type of recruited cells while TNFR2 affects cell function. However due to the effect of TNFR1 on the expression of MHC-II on myeloid cells, the cellular function can be also impaired in the absence of TNFR1.

Both myeloid and lymphoid expression of TNFR1 influence the frequency of NK cells in the liver. Specially, after BCG infection, the frequency of NK cells increases more than two-folds and their recruitment appears to be mediated by TNFR1. NK cells play an important role during mycobacterial infections and also have been implicated in the pathogenesis of liver diseases^36,37^.

We also found a proportion of NKT cells in the liver of both naïve and BCG infected mice and that TNFR1 affects their frequency. However, myeloid or lymphoid TNFR1 expression increases the number of NKT cells compared to TNFR1 KO mice. A previous study has described that Vα 14 NKT cells can play an anti-inflammatory role mediated by TNF in mice infected with BCG as mice lacking Vα 14 NKT cells generated necrotic granulomas and higher mRNA TNF production indicating higher inflammation in the liver that could be controlled by anti-TNF^38^.

TNFR2 has been shown to promote the expansion and function of Tregs which exhibit a CD3^+^CD4^+^CD25^+^FoxP3^+^ phenotype^23,39^. We found that neither myeloid not lymphoid lack of TNFR2 expression modify the frequency of Tregs in the liver. In contrast, lack of TNFR1 in lymphoid cells resulted in lower frequency of Tregs in the liver. However, when the capacity of Tregs from BCG-infected mice were tested, Tregs from TNFR2 T-KO were less efficient in inhibiting CD4 T cell proliferation in association with deficient TGF-β production. These data indicates that although absence of TNFR2 on myeloid or in lymphoid cells does not change Treg frequency of, TNFR2 expression is required for suppressive activity. These data support the hypothesis that TNFR2 can be used as target with pharmacological agents under different diseases to maintain immunological homeostasis^40^.

Myeloid population recruited to the liver after BCG infection was analyzed to clarify the influence of TNFR1 expressed by myeloid or lymphoid cells. We observe that TNFR1 deficiency, on either myeloid or lymphoid cells, affected the frequency of liver macrophages. We were interested in the study of the particular myeloid population expressing CD3 and TCR markers that has been described to form mycobacterial granulomas in both mouse and human livers^19^. The presence of these cells in granulomas was shown to be regulated by TNF as anti-TNF prevents their recruitment or differentiation in granulomas^19,41^. We have identified two subpopulations of CD3^+^ myeloid cells that phenotypically are CD3^+^CD11b^+^TCRαβ^−^ and CD3^+^CD11b^+^TCRαβ^+^. These cells are in the liver of naïve and infected mice and their frequency depends on TNFR1 expression but are independent of TNFR2. A majority of CD3^+^CD11b^+^ are negative for TCRαβ and the proportion of these cells increases by more than 3-folds after the infection. In contrast, CD3^+^CD11b^+^TCRαβ^+^ is a minor population in naïve livers and increases by two-folds in WT infected mice.

The specific role of CD3+ myeloid cells is still unknown, however the growing evidence of their presence in diverse pathologies including malaria infection and cancer suggests their involvement in pathological process^42–44^. As these cells are able to phagocyte, a role in the activation of cells recruited to activate intracellular mycobacterial killing can be proposed but this remains to be elucidated. We also explore whether a macrophage cell line can express lymphocyte markers under infection with BCG and confirmed that at the protein and mRNA levels, CD3 subunits and TNF were upregulated in a time dependent manner. Thus, it is the first report indicating a role for TNFR1 in the recruitment of CD3+ myeloid cells at the infection site which provides new insight into the mechanisms involved in the generation of this subpopulation whose origin and function still remain to elucidate.

In conclusion, tmTNF and solTNF interacting with myeloid cells expressing TNFR1 is the first line of host defense mechanism against mycobacteria and the main TNF signaling mediating liver injury by recruiting mononuclear cells that cannot only protect but also cause tissue damage by secreting cytokines and other pro-inflammatory factors.

## Material and Methods

### Animals

Mouse strains used are: C57BL/6 wild-type (WT), fully deficient for TNFR1 (TNFR1 KO), Tnfrsf1α conditional knockout mice (Tnfrsf1αfl/fl) were crossed to LysM-Cre or CD4-Cre strains to obtain p55 TNFR1 inactivation in myeloid cells (TNFR1-M KO, Tnfrsf1αfl/fl LysMcre/wt), or T lymphocytes (TNFR1-T KO, Tnfrsf1αfl/fl CD4cre) respectively, as previously was described^14^. CD4cre/TNFR2fl/fl mice that do not have TNFR2 on the surface of T cells (TNFR2-T KO) were obtained by crossing C57BL/6NTac-Tg(CD4-cre) (from Taconic farms) with TNFR2fl/fl mice (TNFR2-Flox) (from EUCOMM via Institute Clinique de la Souris, France from Prof Daniela Mannel, University of Regensburg, Germany), as previously was described^15^. Finally, mice that do not have TNFR2 on the surface of myeloid cells (TNFR2-M KO) were obtained by crossing TNFR2fl/fl with LysM-Cre mice gently provided by Prof. Daniela Männel^45^. All mouse strain (WT, TNFR1 KO, TNFR1-M KO, TNFR1-T KO, TNFR2-Flox, TNFR2-M KO and TNFR2-T KO) were maintained under conventional conditions in the animal facilities of the Medical Faculty, University of Geneva, and the Transgenose Institute, Orleans, France. Experiments were done in accordance with institutional guidelines and were approved by the academic ethical committee on experimentation and the cantonal veterinary office from Geneva (GE/211/17 and GE/63/18).

### *M*. *bovis* BCG preparation, infection and CFU

*M*. *bovis* BCG Pasteur strain 1172 P2 (Pasteur Institute, Paris, France) was used and grown to the log phase in 7H9 middlebrook medium supplemented with oleic albumin dextrose catalase (OADC). The bacteria were then harvested, washed, and frozen at −80 °C in PBS plus 10% of glycerol. Bacterial load was determined by plating serial 10-fold dilutions on 7H10 middlebrook agar (supplemented with OADC) and counting colonies after incubation for at least 3 weeks^32^.

Mice were infected intravenously (i.v) with 1 × 10^7^ living *M*. *bovis* BCG strain Pasteur in 100 µL of saline, as previously reported^18^. Mice were monitored twice a week and sacrified 15 days after infection. Groups of uninfected or naïve mice of the different genotypes were killed and liver cells analyzed similarly to infected mice.

Colony forming units (CFU) were performed in liver tissues at 2-weeks post-infection as previously reported^16^.

### Histologic analyses

Lung, spleen and liver were fixed with 4% paraformaldehyde, processed, and embedded in paraffin. Sections (5 µm) were stained with hematoxylin and eosin (H&E) or Ziehl-Neelsen (ZN). Histopathological analysis were performed on tissue sections from liver using digital microscopic images acquired by a Zeiss Axioscan.Z1 and analyzed with ZEN 2 software (Carl Zeiss Microscopy). For liver analysis, the extend of lesions was quantified and is presented as the percentage of the area with lesions, corresponding to the area of granulomas/surface area of the total liver.

### Isolation of liver mononuclear cells (LMCs)

LMCs were obtained by a modified collagenase digestion method based on the previously reported method^46^. Briefly, mouse liver was perfused with Hank’s buffered salt solution containing 0.025% collagenase, removed and passed through 70-µm strainer. Cell suspension that was resuspended in 40% Percoll was overlaid onto 70% Percoll and centrifuged at 750 g for 20 min. LMCs were collected from the interface.

### RAW cell culture

The murine macrophage cell line RAW 264.7 (RAW macrophages) was maintained in DMEM supplemented with 10% head-inactivated FBS, penicillin, streptomycin, sodium pyruvate, glutamine, and HEPES (complete DMEM) at 37 °C in a humidified atmosphere containing 5% CO_2_.

### BCG *In vitro* infection

LMCs, CD3^+^CD11b^+^TCRαβ^−^, CD3^+^CD11b^+^TCRαβ^+^ and RAW cells were obtained as described. LMCs, CD3^+^CD11b^+^TCRαβ^−^ and CD3^+^CD11b^+^TCRαβ^+^ were infected with BCG Pasteur (multiplicity of infection [MOI], (1) by 24 hours (h). RAW macrophages were cultured in 24-well flat-bottomed cell culture plates (1 × 10^6^/mL) and infected with BCG Pasteur at MOI 0.5 along 2, 5, and 24 hours (h) at 37 °C in a humidified atmosphere containing 5% CO2. Cultured cells were maintained with DMEM supplemented with 10% head-inactivated FBS and antibiotic free medium. Supernatants were used to measure cytokines.

### Multiparametric cytometry analysis

Single-cell suspension from LMCs was prepared, the cellular phenotype was determined by flow cytometry. The following monoclonal antibodies were used: Gr-1, CD11b, Ly6C, F4/80, CD11c, MHC-II, CD2, CD3, TCRαβ, CD4, CD8, α-GalCer mouse CD1d complex (L363), NK1.1 (PK136), FoxP3 and CD25 (Biolegend). Briefly, LMCs were suspended in PBA and stained with antibody cocktail from 30 min at 4 °C and washed with PBA. To determine the intracellular expression of FoxP3, LMCs were fixed (fixation buffer; Biolegend) and permeabilized (Perm/Wash buffer; BD Pharmingen, San José, CA, USA). After permeabilization, cells were washed twice and then stained with anti-human perforin mAb. Cells were washed and resuspended in 1% paraformaldehyde.

The percentage of RAW macrophages expressing CD3 and transmembrane TNF (tmTNF) was assessed by flow cytometry, following the same protocol to LMC’ cellular phenotype.

Fluorochrome-labeled isotype-matched control antibody was used to evaluate background staining. After incubation with antibodies, cells were washed twice in PBA and data collected using a FACs CyAn and analyzed with FlowJo software. 100,000 events were acquired per sample.

### Enrichment of regulatory T (Treg) cells

LMC suspensions were obtained as previously was described from the liver of infected mice. Treg were enriched by using magnetic microbeads kit (CD4+ CD3+ Regulatory T cells isolation kit; Miltenyi Biotec) and AutoMACS Pro Separator (Miltenyi Biotec), following the provider instructions. First, CD4+ T cells were pre-enriched by depletion of unwanted cells, non-CD4+ T cells were indirectly magnetically labeled with a cocktail of biotin-conjugated antibodies and anti-biotin Microbeads. Then, CD25+ cells were positively selected from the enriched CD4+ T cells.

### Enrichment of CD4+ T cells

Spleen cells from uninfected WT mice were prepared as described previously^32^. CD4+ T cells were isolated from splenocytes by negative selection using magnetic cell sorting technology (CD4+ T cells isolation kit; Miltenyi Biotec) and AutoMACS Pro Separator (Miltenyi Biotec). Non-CD4+ T cells were indirectly magnetically labeled with a cocktail of biotin-conjugated antibodies and anti-biotin Microbeads and negative fraction was CD4+ T cells.

### Cell stimulation and proliferation assays

To assess the functional activity of Tregs, CD4+ T cells from uninfected WT were stimulated with anti-biotin MACSiBead particles, biotinylated antibodies against CD3 and CD28 plus anti-biotin particles (T cell activation/expansion kit; Miltenyi Biotec), and 50 IU recombinant IL-2. MACSiBead particles were used at a ratio 1:2 (cell-to-bead). Tregs were co-incubated with varying ratios of CD4+ T cells (CD4+ T cells-to-Treg, 1:1, 2:1, 4:1), and after 72 h of co-culture at 37 °C, supernatants were collected for cytokine measurements and cells for proliferation assay using KI-67 (Clone 16A8) (Biolegend). Briefly, cells were harvested, washed with PBA, and then cell pellet suspended in Fixation/permeabilization solution (eBioscience) at 4 °C, washed with permeabilization buffer (eBioscience), and stained with KI-67 for 30 min at 4 °C. Cells were washed and analyzed by flow cytometry. CD4+ T cells with anti-biotin MACSiBead particles and without Treg, were used as polyclonal stimuli and considered as 100% proliferation (Positive control).

### Sorting of cells

To sort CD11b+ CD3− and CD11b+ CD3+, or CD3+ CD11b+ TCRαβ−, and CD3+ CD11b+ TCRαβ+ cells, single cell suspensions were obtained from liver of WT, TNFR1 KO and TNFR2-T KO after 2-weeks BCG infection. Cells were stained with monoclonal antibodies against CD11b, CD3 and TCRαβ+ for 30 min at 4 °C. Thereafter, the cells were sorted on a FACS Aria II (BD Biosciences), using the following strategy: doublets were gated out by FSC-A vs. FSC-H and dead cells were excluded by analyzing the negative region on the diamidino-2-phenylindole (DAPI) dye. Using double stained to CD3 and CD11b in order to sort individually CD11b+ CD3+ and CD11b+ CD3−. To obtain CD3+ CD11b+ TCRαβ−, and CD3+ CD11b+ TCRαβ+, a first CD3+ gate was limited, then double stained cells with CD11b and TCRαβ were evaluated in order to delimit the adequate gate.

### Real-time PCR for TNF, CD3e, CD3z and CD2

For the analysis of TNF, CD3e, CD3z and CD2 gene expressions, total RNA from 5 × 10^5^ cells (RAW) and 50’000 cells (Liver cells) were isolated using TRIzol Reagent (Invitrogen) following the manufacturer’s protocol. RNA was eluted in 12 µL of nuclease-free water and the quantity of extracted RNA was evaluated using NanoDrop 2000c spectrometer (ThermoFischer Scientific). A total of 500 ng of total RNA was converted to cDNA using iScript cDNA Synthesis Kit (Bio-Rad, Switzerland) as per the manufacturer’s guidelines. Gene expression analyses was performed with the 7500 Real-Time PCR Systems (Applied Biosystems) using standard thermal cycling conditions and SYBR green assays specific for mTNF-a (F: CCACCACGCTCTTCTGTCTA, R: AGGGTCTGGGCCATAGAACT), mCD3e (F: CTGGTGCCTTCTTCAGAAATG, R: AGGATGCCCCAGAAAGTGTT), mCD3z (F: ATCCCAGGGAAGCAGAAGAT, R: AGAGTTTGGGATCCAGCAGA), and CD2 (F: TCTGCTCTTCAGCCTTTCCG, R: CTCCTTACCCATCGCACCTC). Data were normalized to two endogenous controls, Glyceraldehyde 3-phosphate dehydrogenase (GAPDH) (F: TCCATGACAACTTTGGCATTG, R: CAGTCTTCTGGGTGGCAGTGA) and hypoxanthine guanine phosphoribosyltransferase (HPRT) (F: GCTCGAGATGTCATGAAGGAGAT, R: AAAGAACTTATAGCCCCCCTTGA). Before gene expression analysis, cDNA samples were serially diluted to 1:2, 1:4 or 1:16 and 2 µL were used as template for the quantitative real-time PCR (qPCR) to perform the validation of the delta-delta CT method. Same cDNA dilutions were used for the all qPCR assays, and relative gene expression values of all different gene targets were calculated using the 2-DDCT formula. The expression of each target gene is presented as the relative expression of the gene. All the qPCRs were run in triplicate along with no-template controls.

### Enzyme levels in serum

Blood samples were obtained from retroorbital sinuses to evaluate hepatocyte damage by measuring serum enzyme activities of aspartate aminotransferase (AST) and alanine aminotransferase (ALT) using an automated procedure (Cobas 8000, Roche) as previously reported^18^.

### Statistical analyses

Data are shown as mean +/− Standard error of the mean (SEM). Multiple *t* test correct for multiple comparison using the Bonferroni-Dunn method were to compare more than two groups. Statistical analyses were performed with GraphPad Prism software (GraphPad Soft., La Jolla, Calif.). *P* value < 0.05 was considered as statistically significant.

## Supplementary information

Data set 1, data set2, data set 3, dataset 4, data set 5, data set 6, data set 7
