# Supplementary material for: Myeloid cell TNFR1 signaling dependent liver injury and inflammation upon BCG infection
Source: Sci Rep. 2019 Mar 28;9:5297. doi: 10.1038/s41598-019-41629-9 (PMC6438980; doi:10.1038/s41598-019-41629-9)
Supplement: Supplementary file 1 — Data set 1, data set2, data set 3, dataset 4, data set 5, data set 6, data set 7 [file 41598_2019_41629_MOESM1_ESM.pdf]

Supplementary data

Myeloid cell TNFR1 signaling dependent liver injury and inflammation upon BCG infection

Leslie Chavez-Galan, Dominique Vesin, Guillaume Blaser, Husnu Uysal, Sulayman Benmerzoug, Stéphanie Rose, Bernhard Ryffel, Valérie F. J. Quesniaux, and Irene Garcia

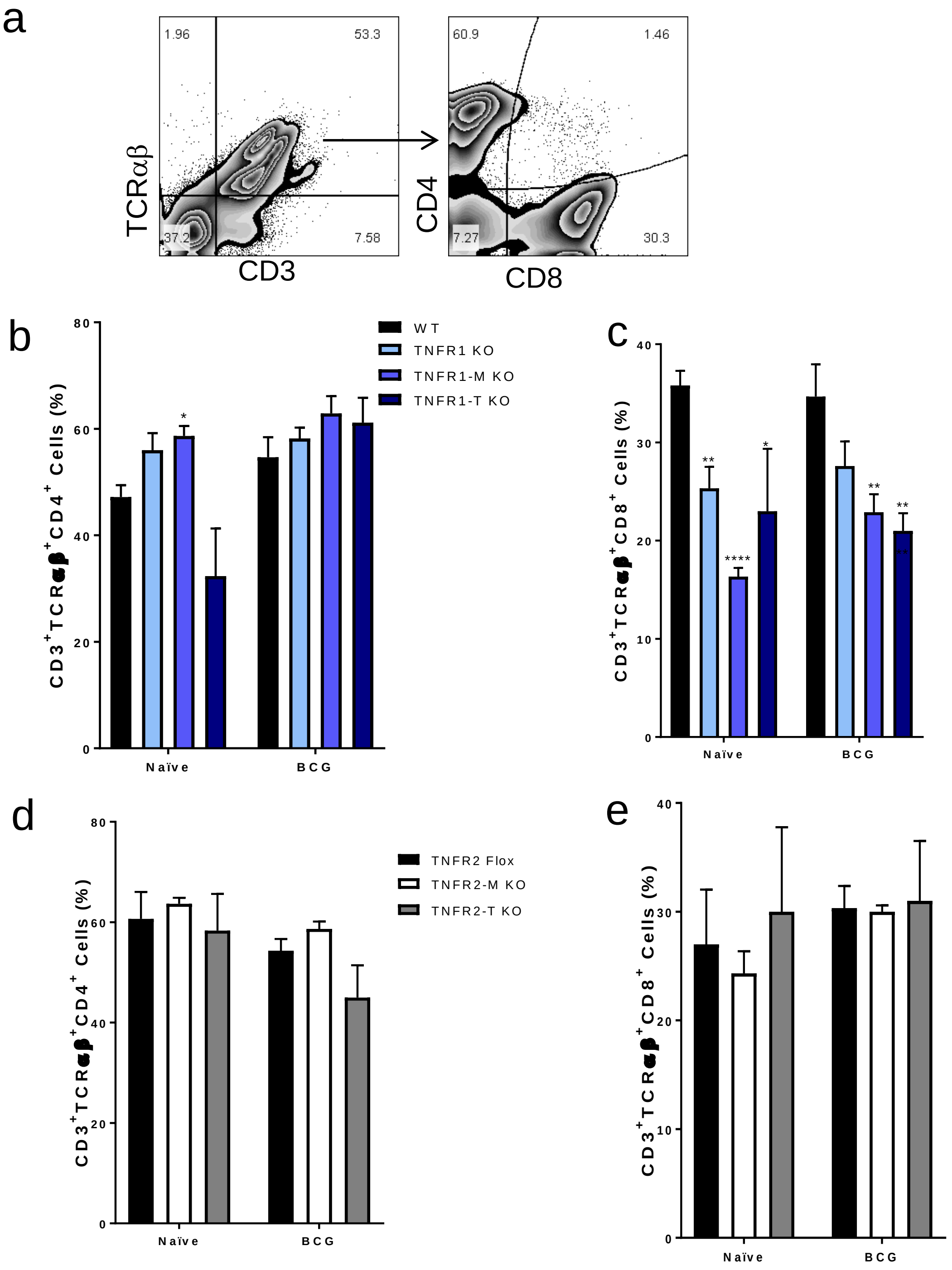

Supplementary Fig. S1. CD8+ T cell recruitment is affected by depletion of TNFR1 in BCG-infected mice. (**a**) Representative zebra plot showing liver cells from WT mice at 2-weeks infection, we delimited the gate of CD3+TCRαβ+ (T cells) and inside this gate evaluated the expression of CD4 and CD8. (**b** and **c**, respectively) The frequency of the subpopulations CD4 and CD8 T cell in naïve littermates and at 2-weeks post-infection of WT, TNFR1 KO, TNFR1-M KO, TNFR1-CD4 KO. (**d** and **e**, respectively) The frequency of the subpopulations CD4 and CD8 T cell in naïve littermates and at 2-weeks post-infection of TNFR2-Flox, TNFR2-M KO and TNFR2-T KO. Results are representative of three independent experiments. Bar graphs shown mean +/- SEM (B-G). n= 3 to 9 mice per group. *p<0.05, **p<0.01 versus WT, Multiple t test and Bonferroni-Dunn were used to multiple comparison.

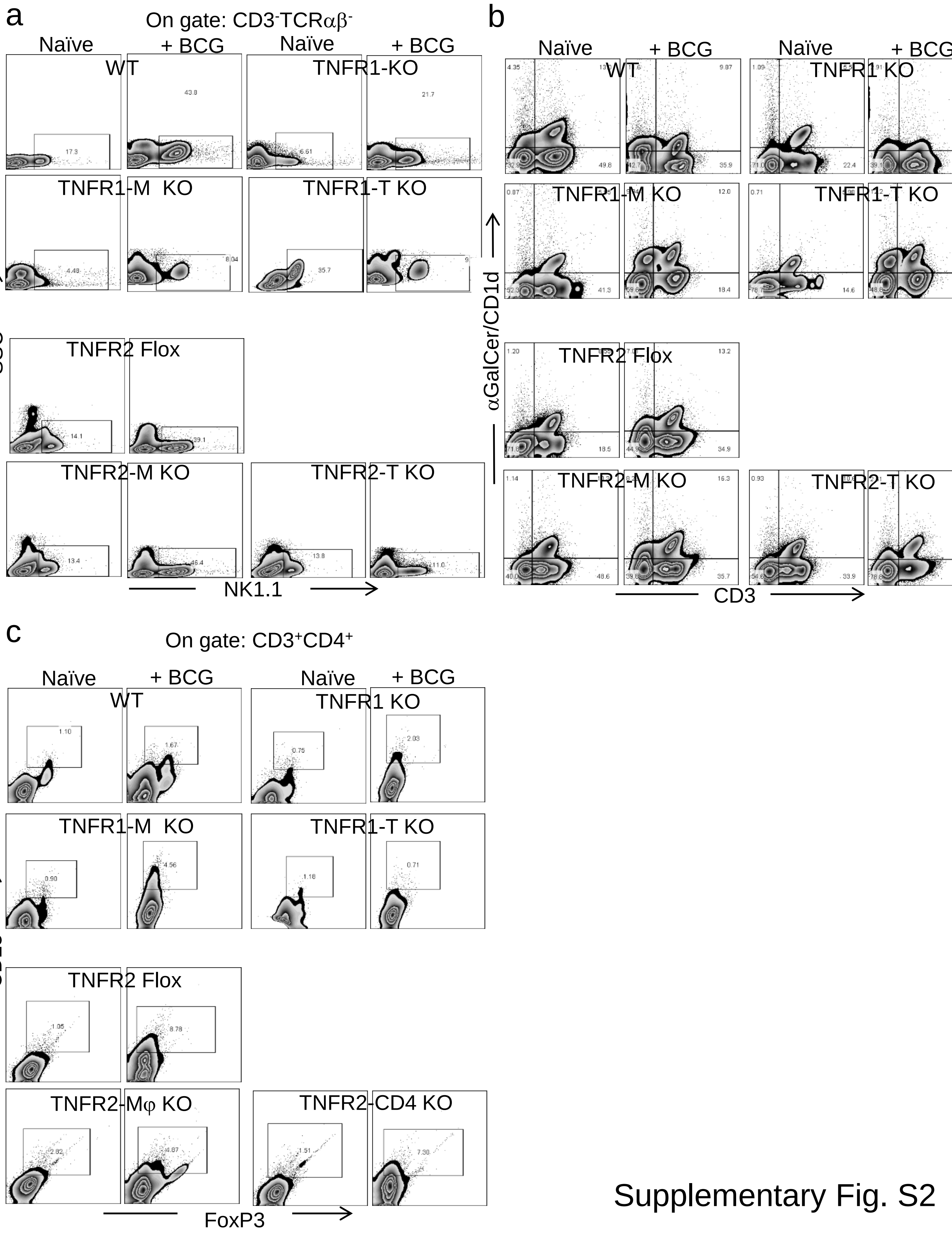

Supplementary Fig. S2

Supplementary Fig. S2. Strategy of analysis to characterize NK, NKT and Tregs cells. Liver cells from WT, TNFR1 KO, TNFR1-M KO, TNFR1-T KO, TNFR2-Flox, TNFR2-M KO and TNFR2-T KO mice by flow cytometry. Representative zebra plot showing liver cells from naïve littermates at 2-weeks infection post-infection. (**a**). To identify NK cells, we delimited the gate of CD3-TCR$\alpha\beta$- and inside this gate, evaluated the expression of NK1.1 (**b**). NKT cells evaluated as double positive cells for CD3 and the tetramer αGalCer/CD1d. (**c**). To characterize Tregs cells, we delimited in the gate of CD3+CD4+ and inside this gate, evaluated the expression of CD25 and FoxP3

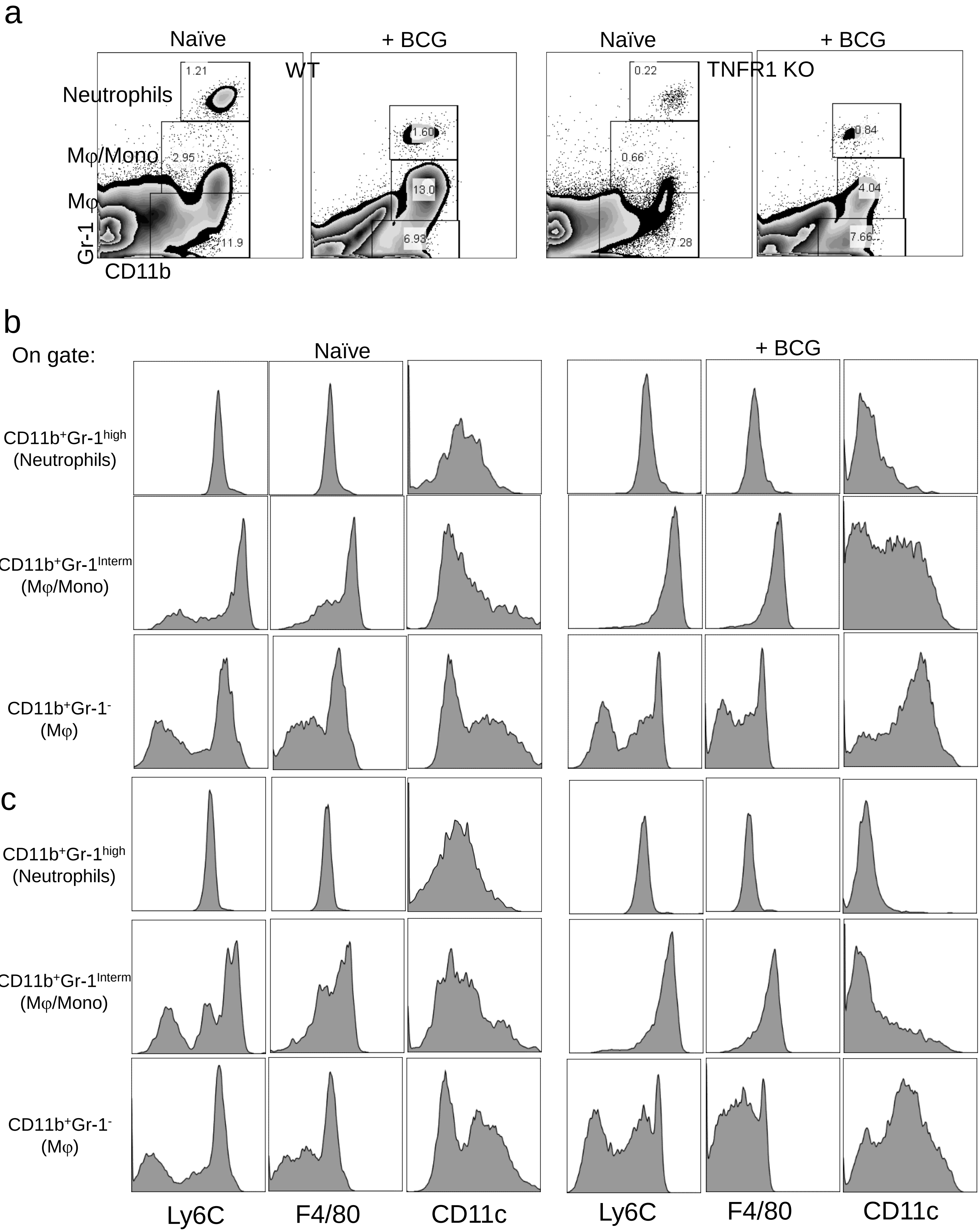

Supplementary Fig. S3

Supplementary Fig. S3. Phenotypical characterization of myeloid cells among mononuclear liver cells.(**a**) Representative zebra plot showing liver cells in naïve littermates and at 2-weeks infection from WT and TNFR1 KO mice, liver myeloid subsets were identified by CD11b and Gr-1 expression. (**b**) Inside the gate of Neutrophils ($CD11b^{+}Gr\text{-}1^{high}$), Mφ/Mono ($CD11b^{+}Gr\text{-}1^{Interm}$) and Macrophages (Mφ) ($CD11b^{+}Gr\text{-}1^{-}$) the expression of Ly6C, F4/80 and CD11c were evaluated for both WT and TNFR1 KO mice (**b** and **c**, respectively). Results are representative of at less three independent experiments.

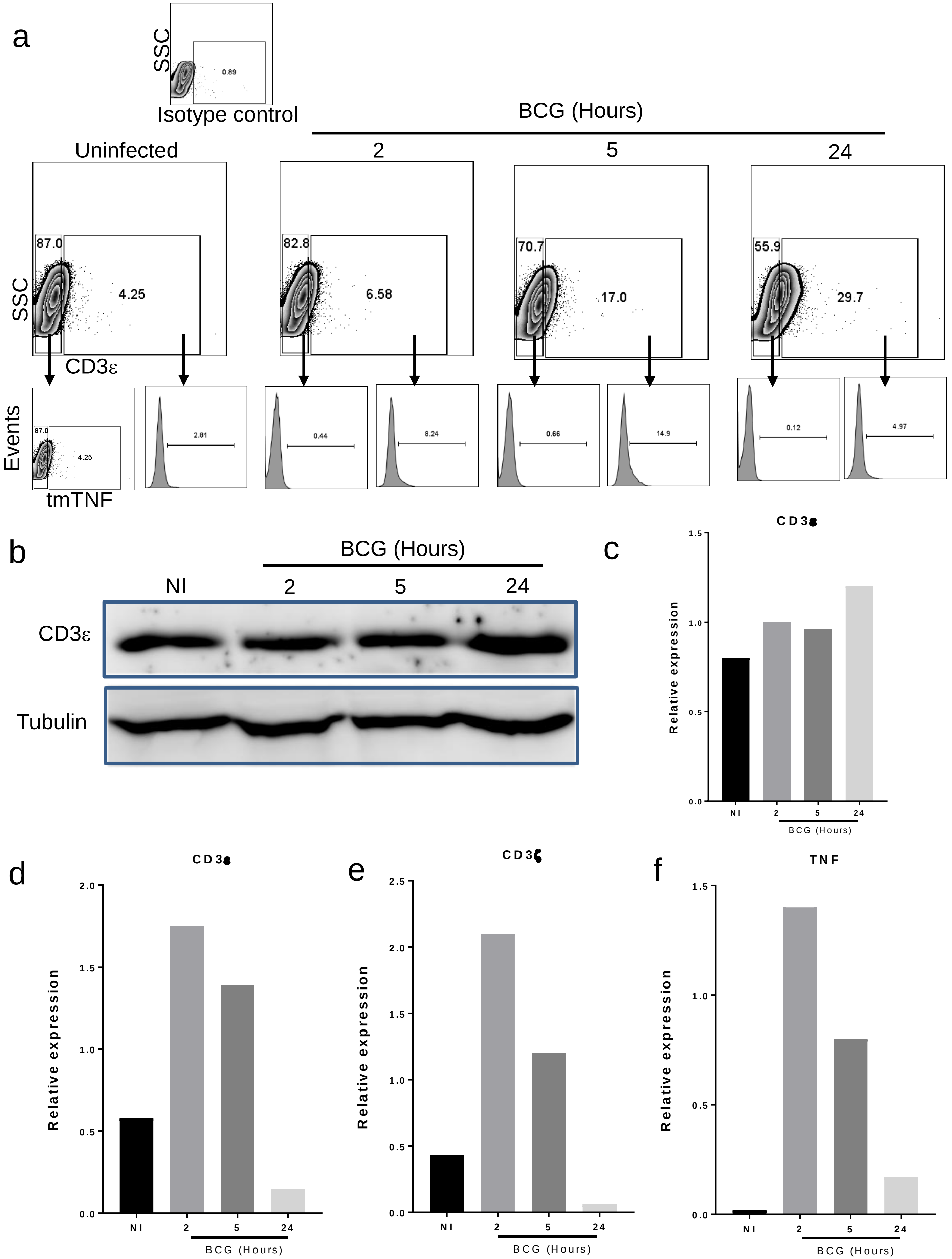

Supplementary Fig. S4

Supplementary Fig. S4. BCG infection increases CD3 and TNF expression at the protein and transcriptional level in RAW cells. (**a**) RAW cells were infected with BCG MOI 0.5 during 2, 5 and 24 hours, and prepared to flow cytometry, western blot and RNA extraction. Representative zebra plot showing CD3ε expression and histograms display the mean fluorescence intensity (MFI) of TNF inside gate CD3- and CD3+. (**d**, **e** and **f**, respectively). (**b**) Protein from infected RAW cells prepared for western blot. Representative western blot for CD3ε and tubulin as loading control. (**c**) Band density of CD3ε normalized with tubulin by densitometry RNA was obtained from BCG-infected RAW cells, the CD3ε, CD3ξ and TNF relative gene expression was evaluated by real-time PCR. Figure representative of 3 independent experiments.

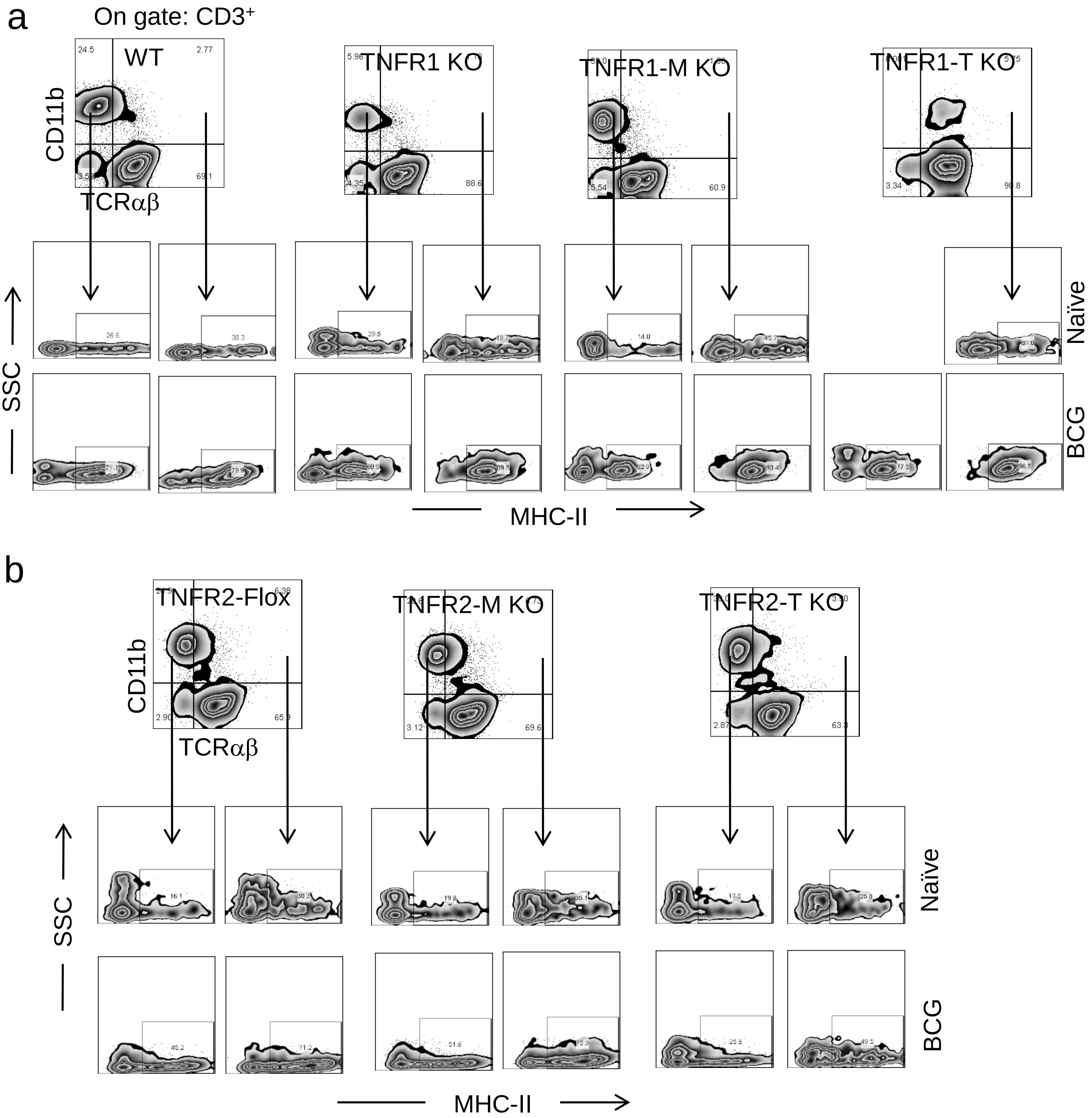

Supplementary Fig. S5. MHC-II expression on CD3+CD11b+TCRαβ- and CD3+CD11b+TCRαβ+ cells in liver cells. (a) Representative zebra plot showing liver cells in naïve littermates and at 2-weeks infection. Previous gate for CD3 was done, posteriorly, CD11b and TCRαβ was identified, inside gate for CD3+CD11b+TCRαβ- and CD3+CD11b+TCRαβ+ cells frequency of MHC-II+ cells was evaluated in WT, TNFR1 KO, TNFR1-M KO and TNFR1-T KO or (**b**) TNFR2-Flox, TNFR2-M KO and TNFR2-T KO mice. Results are representative of at less three independent experiments.

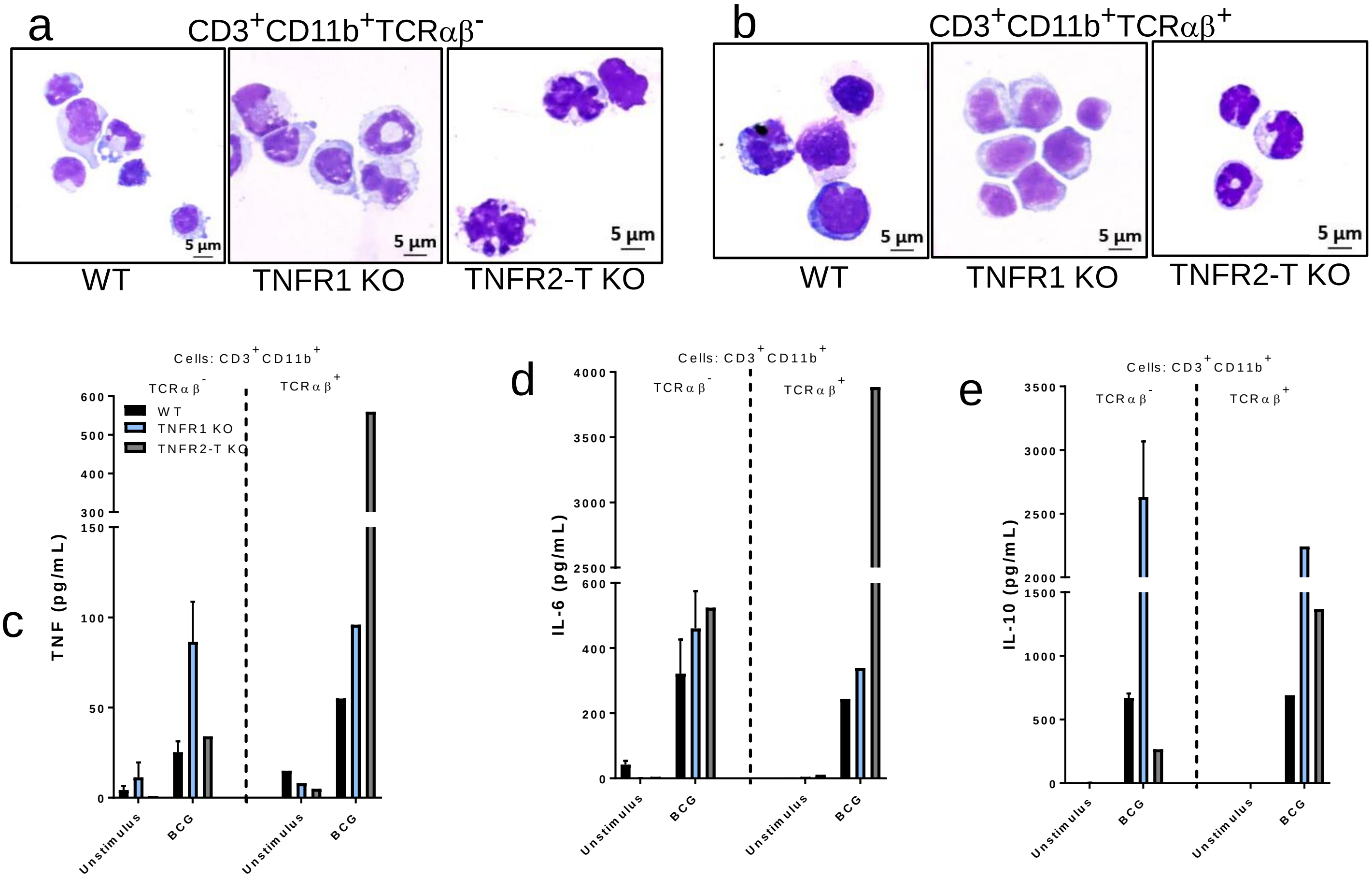

Supplementary Fig. S6. CD3+ myeloid cells are able to produce cytokines after re-activation with BCG. (**a** and **b**) Liver cells from WT, TNFR1 KO and TNFR2-T KO were obtained and prepared for sorting. Sorted by flow cytometry CD3+CD11b^{+}TCRαβ- and CD3+CD11b^{+}TCRαβ+ were used for cytospin, and photomicrographs from cytospin preparation stained with May-Grüenwald-Giemsa (to show the morphology. (**c**, **d** and **e**, respectively). CD3+CD11b^{+}TCRαβ- and CD3+CD11b^{+}TCRαβ+ sorted cells were restimulated with BCG MOI for 1 36 h, and TNF, IL-6 and IL-10 were measured in the supernatant. A pool of liver cells was used from individual genotypes (WT 2 mice, TNFR1 KO 4 mice and TNFR2-T KO 3 mice), after 2-weeks post-infection.

Supplementary Fig. S6

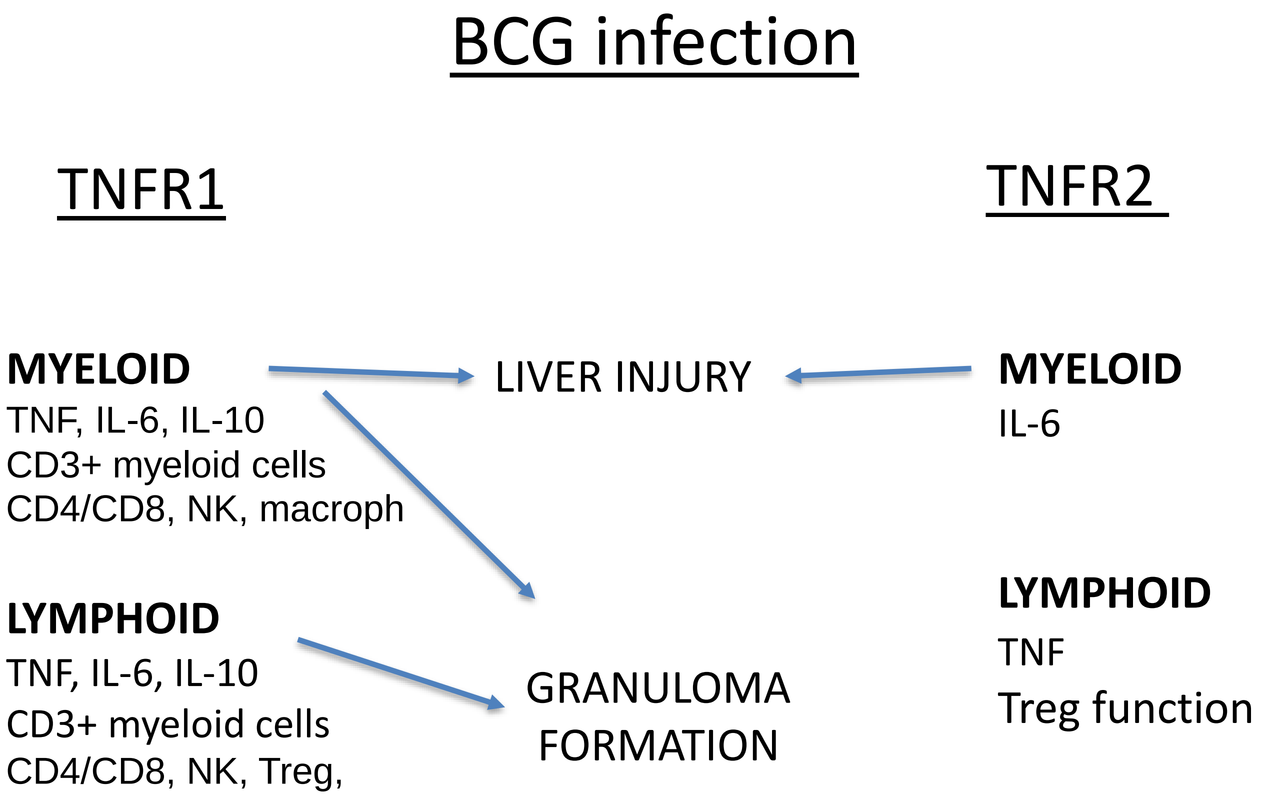

Supplementary Fig. S7. Schematic representation of the effects of TNFR1 or TNFR2 cell specific expression on myeloid or on lymphoid cells. Myeloid cell TNFR1 plays a role in both liver injury and cell recruitment to form granulomas. Lymphoid cell TNFR1 only plays a role in granuloma formation. Myeloid cell TNFR2 influences liver injury mediated by IL-6. Lymphoid cell TNFR2 plays a role in Treg function.

Supplementary Fig. S7
